# President’s Address

**Published:** 1908-08-15

**Authors:** Oel E. Lanphear

**Affiliations:** Paw Paw, Michigan


### PRESIDENT'S ADDRESS.

BY DR. OEL E. LANPHEAR, PAW PAW, MICHIGAN.

Read before the Southwestern Michigan Dental Society, April 14 and 15, 1908

A most pleasant place to meet is this fair city, perfect type of Michigan's industrial centers, of first rank in furniture making the world over, embellished by nature's choicest gifts and abounding in generous hospitality and hearty good-will. The cordial welcome we have received together with the care and precision with which the zealous local committee has worked, give ample promise of an enthusiastic meeting. Many will appreciate our program, but few realize the energy and time required to produce it. I wish to take this occasion to publicly thank all who have so kindly contributed to the success of this meeting, be he officer, committeeman or member.

This great profession of ours is in a state of intellectual unrest; the majority are ever striving to increase their intellectual grasp and to stimulate their mental digestion.

This striving finds expression in the multiplicity of dental meetings held and the volume of dental literature published annually. These meetings "are the clearing houses" of advanced professional friendships. They help to encourage the formation of many permanent professional friendships which aside from social enjoyment, serve to reduce friction, rivalry and dissension. Here, wide-awake men may meet their neighbors, grasp them by the hand, look into their faces and compare investigations, experiences and opinions by face to face discussion. Here the great and near-great need not seek for personal aggrandizement. Everything is arranged for the benefit of all practitioners, in-so-far as they care to partake of the good things offered. The inspiration of your presence is most earnestly desired as well as any suggestions you may offer to help some brother practitioner to secure better results from his work. This reacts by producing a marked benefit, not only directly to ourselves, but indirectly to our clientele. Dental meetings provide most excellent opportunities for the reciprocity of ideas. The vigilant stand ready to adopt the best means and methods and raise the standard of professional ideals. They who would achieve professional ideals must be business men and make a business of their profession. They must strive for "broader and deeper intellectual training not simply in the theoretical and practical, but in all branches of knowledge which strengthen the mind."

A powerful stimulus is gradually bringing about a unity of action throughout the profession, making possible, not only successful district and local meetings, but great national and international gatherings. Concentration and co-operation will eventually bring about the amalgamation of the profession into one great world-unit. All the great institutions known to men are builded upon concentrated effort. A universal wave of organization is sweeping over the dental world. All men conversant with existing conditions realize the necessity for reciprocity and interstate licensing boards, whose requirements and examinations are practically the same. The state boards should be given power to register approved candidates. A strenuous individual effort should be put forth to cause every ethical dentist to feel the necessity of attending the meetings of his local and state societies. A membership in the local society should carry with it a membership in the next higher organized unit. Thus a national association might be built up of sufficient strength to bring about active uniform dental legislation, maintain and develop vital relations with their constituency and to publish a weekly journal carrying to every member a concise report of the new, and an authentic history of the old, making it possible that an operation originating in Berlin or London may be put in practice in New York or San Francisco within a fortnight. The National Association has voted to publish such a journal, and has an active committee working on the details. A guaranteed circulation is one of the requirements for the publication of this associational organ, and this can be secured only by developing the strength of the association until it reaches proportions adequate to the task. The time is not far distant when a paid membership in the National Dental Association will carry with it a subscription to the best journal in existence. The Southwestern Michigan Dental Society, aside from its good work, will be strengthened by a just consideration of this great organization movement. We need more earnest and zealous workers in our local societies, men who are willing to work for the uplifting of their societies, regardless of personal feelings and prejudices. Doctor Peck, in his address at Minneapolis, says: "In-so-far as our purpose centers in the welfare of the profession we represent, in-so-far as we seek only the good of the association, our striving is laudable. When we forget the good of the brotherhood in aims that are purely personal and selfish, we dishonor ourselves and do untold injury to the cause we claim to cherish."

Until each individual dentist becomes aroused from the accursed apathy which has so long threatened the profession and become alive to the great truth that only by his earnest human effort can the best good be accomplished, will perfect amalgamation and consolidation be attainable.

One of the most deplorable conditions in existence in our work to-day is the lack of training of the youth of our land in the proper use of the teeth and care of the mouth. Are we going to leave this most important phase of our work to be carried on by the firms engaged in the manufacture of proprietary dental adjuncts, under the semblance of "Educational Associations"? Rather, let us condemn such work and set ourselves the task of removing the possibility of such so-called "Educational" work being carried on in this way. I would recommend that our society endorse the work of well-trained, efficient dentists, as our Dr. A. C. Runyan, who has made the presentation of this branch of our work a study for years, and encourage trained men to appear before the teachers' associations, mothers' clubs and social and literary organizations of America, for the purpose of enlightening the present generation and preparing the way for the education of generations to come by securing a place for oral hygiene in the curriculum of the public schools.

Let us stand by the best of intention, attention and retention. May our intention be to look for the best in others and give the best we have to fill our niche to accomplish our task, to leave the world better than we found it, in short, to make our lives an inspiration to the many. May our attention be concentrated upon finding the largest measure of happiness in the cultivation of thoroughness of work, kindness of heart and gentleness of manner, upon the admiration of earth's beauty and comfort in the promises of Divine love.

May our retention find realization in successful fillings, and successful aims adhered to throughout our professional careers, admitting of naught but the best motives and actions, our reward achieved in the thought of having done well in all things attempted.

He who has done all these things has achieved success and holds the whole country under obligation.

DISCUSSION

Dr. N. S. Hoff: I would like to say some very complimentary things about the address of the president. I could not say anything else. It was a very concise and truthful statement of the professional attitude towards our work to-day. The value of society work is one that I believe you are to have up for discussion later in the session. This is one of the most important things that there is in our dental atmosphere just now; what shall we do, and what are we to gain by a closer organization? It is a vital subject with us just now, and one that we ought to discuss calmly and seriously, and do what we can to bring this matter to some satisfactory conclusion.

The subject of dental reciprocity, or an interstate license, is also a very large and important subject. It is one that has had a great deal of discussion in recent years, and one that will have a great deal more in the years to come, as it is not yet settled. We have a provision in our new state law that gives us a basis for interstate reciprocity as far as we are concerned as a state, and I don't know that we can discuss it from any other standpoint than our own legal status. It is, unfortunately, a very difficult matter to adjust satisfactorily on a legal basis. On a legal basis things have to be adjusted so and so, and one cannot make allowance for anything or anybody. Whether it is a matter of equity or professionalism we cannot take into consideration strict professional ethics in the adjustment of these interstate or international relations of professional men. I think it is almost deplorable that we have to resort to legal measures to establish our professional standing. It is not the best way in my judgment. We ought to establish our relations upon an educational basis, or on a professional basis, if you please, which means an educational basis. If we could do so, it would put our state board in a position to handle the subject professionally, and in a way that would be entirely satisfactory. We have the idea in our minds that we must establish these interstate relations on a legal basis and I don't see any use of our discussing it on any other basis. It is the only way that in the very near future we are going to be able to consider this question at all. We may talk all we please about professional courtesies and the desirability of establishing reciprocity on professional grounds, but we shall not be able to do it, at least not for some time to come.

The president brought up another important matter that I want to say just one word about; it is the matter now very prominently before the professional public at least—and is being forced by the profession to the notice of the general public. It is the education of the public in the matter of dental hygiene, through our public schools and eleemosynary institutions, and through the more recent propaganda of certain commercial institutions. I don't believe any of us sympathize at all with the idea of doing this work through commercial institutions of any sort. I think as a profession it belittles us to sanction such enterprises. It is not on a right basis in my judgment, and although we may try to excuse ourselves on the plea that the end justifies the means, I don't believe that we will respect ourselves as a profession as much if we should undertake to do the work in the unethical way, as we will if we shall be compelled to do less work, and make less progress by a more truly professional course. A great many people have objected to taking Mr. Carnegie's money and Mr. Rockefeller's money for school purposes and for libraries, because it is tainted money. I don't think anybody's money is tainted so long as it is current, but it seems to me we are in an entirely different position. We are a profession that is supposed to rest on the basis of honor and public integrity one to another, and that same fraternal integrity is the basis of our relations to the general public. We must consider all of our actions on that basis and if there is an objection to using tainted money from a commercial house, as in this particular case to which the president referred—he didn't specify the particular house, but I think we all know to whom he referred—we have no business, it seems to me, to be playing with that kind of fire. It is one of those things that will react on us, and not to our credit. I most cordially commend this particular phase of the president's address, and say that we should be very careful as to what we endorse in this direction. We should not commit ourselves to this kind of propaganda until we have more seriously discussed and considered its bearing upon our relations to the public, than we have done heretofore. I simply make this statement and don't wish to enforce it or to specify any more particulars about it, but I want to express my own view of all such matters.

DR. M. L. WARD: I am sure the president has gathered a great deal from our current literature regarding the need of reorganization and the benefits to be derived from it. I am glad he has put it before you in the way he has, as he understands so well the benefits that are to be derived and the steps that are necessary to lead up to a better organization than we now have, not only in our local but state and national organization as well. To get any good thing we need unity as well as enthusiasm, and you must get together and agree to do this thing and then heartily co-operate to accomplish the object for which we aim. The spirit of the new organization movement is one grand campaign for members of our societies, local, state and national. Statistics show that in both the medical and dental professions that only 50 per cent of the profession, in the best organized states in the Union, belong to societies. Four years ago in the state of Illinois only 14 per cent of the men registered in dentistry belonged to a society, while to-day a little over 50 per cent belong. One grand campaign has raised the percentage from 14 to 50. In the medical profession the percentage has been raised from about 32 to 55. After six years of hard work they have secured only 55 per cent; even this has accomplished worlds of good. In the dental profession in this state we make a better showing than most of the other states; we have about 25 or 26 per cent of the dentists belonging to our dental societies. We ought to raise this percentage from 25 to what others have, 50 or 60 per cent. In order to induce this percentage of men to attend our dental societies we must have a better organization, we must perfect a plan organization first. In order to perfect such an organization we must have the co-operation of all the now existing societies. The reorganization committee of the state society has adopted the Illinois plan and has successfully put it into effect in a large part of the state. It is a plan which will show results, it has shown results in other places and in other states and will certainly show results here. The time has come when no man that cares to be a professional man can afford to let two or three dollars, or any other ambitions that he may have, stand in the way of his taking an active part in society work. The organizations of labor, in which there is little education and no such general intelligence as we claim, have developed men and measures to a greater extent than we shall if we don't get busy right away. They have perfected an organization that is little to be improved upon, to say the least. We hope that with the assistance of the already existing societies in this state to accomplish some things in the line of concerted professional activity. We are first hoping to get our organization completed, and then to make a grand campaign for members. We cannot do this successfully until the whole organization is perfected.

Dr. B. J. Cigrand: I want to say just a few words. Personal contact at a meeting of this kind does us more good than we realize. Dentists, as a usual thing, have an idea that they can read the proceedings of a society when they come out in the journals. I want to say, and I want to emphasize it, that the proper way to get the inspiration of a paper is to hear the man read it who is the author of it. You can get much out of a paper by reading it at home, and studying it; but after all there is a great deal to be gained from the personality of the author that you derive only when you hear and see the man. I have nothing at all to offer on this general subject at this time, except that I am always enthusiastically in favor of the men of our profession meeting in convention to exchange opinions. There is nothing for debate on this subject, as the president has offered it, that I can see. Organization is essential, and union of effort is everywhere recognized as practical accomplishment. I can only say that when the proper time comes I hope that you will take the proper course which will secure the success of all the state and local societies of Michigan.

